# Seasonal Influence on the Impact of Human Chorionic Gonadotropin (hCG) on Testicular Haemodynamics and Hormonal Responses in Canines: A Comparative Study

**DOI:** 10.1002/vms3.70117

**Published:** 2024-11-13

**Authors:** Behnam Nadali, Arman Abdous, Nima Karami, Mohammad Jokar, Ourang Ataie Amarloie, Mehran Farhoodi

**Affiliations:** ^1^ Department of Clinical Sciences Faculty of Veterinary Medicine Karaj branch Islamic Azad University Karaj Iran; ^2^ Faculty of Veterinary Medicine Calgary Canada

## Abstract

Human chorionic gonadotropin (hCG) is widely used to treat reproductive dysfunction by enhancing testicular blood flow and stimulating hormonal activity. This study investigates the seasonal variations in the response to hCG treatment in male dogs, focusing on its effects on testicular blood flow and plasma concentrations of testosterone and oestrogen. Conducted across different seasons (spring, summer, fall and winter), the study utilised colour Doppler ultrasonography to measure testicular haemodynamics and analysed hormonal levels at multiple time points post‐hCG injection. The findings revealed that the response to hCG is modulated by seasonal factors, with significant variations in both blood flow and hormone levels. A significant negative relationship was indicated between testicular blood flow and testosterone levels, particularly during spring and summer. These results suggest that seasonality should be considered when administering hCG for reproductive treatments in canines.

## Graphical abstract

This study explores the seasonal influence of human chorionic gonadotropin (hCG) on testicular blood flow and hormonal responses in male dogs. Seasonal variations significantly affect both testicular haemodynamics and hormone levels post‐hCG treatment, with spring and summer showing greater improvements in blood flow and heightened testosterone and oestrogen responses. These findings emphasise the importance of considering seasonality when administering reproductive treatments in canines, optimising timing for enhanced fertility outcomes

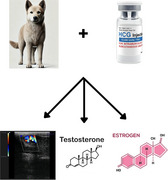

## 1 Introduction

Testicular function is critical for male fertility, with blood flow to the testes playing a vital role in maintaining healthy spermatogenesis and hormone production (Mason 2023). Reduced testicular blood flow can lead to conditions such as hypoxia, resulting in impaired spermatogenesis and testicular atrophy (Tesi et al. 2020). Human chorionic gonadotropin (hCG) is commonly used to enhance testicular function by stimulating Leydig cells to produce testosterone, thereby promoting spermatogenesis and improving overall testicular health (Zdunczyk and Domoslawska 2022).

Colour Doppler ultrasonography has emerged as a valuable tool for non‐invasive assessment of testicular blood flow, providing insights into the haemodynamic changes that accompany various reproductive treatments (Gloria et al. 2020). However, the influence of environmental factors, particularly seasonality, on the efficacy of hCG treatment has not been extensively studied in canines. Seasonal variations are known to affect reproductive physiology in many species, influencing factors such as testicular size, sperm production and hormone levels (Beltrán‐Frutos et al. 2022).

This study aims to explore the seasonal modulation of hCG's effects on testicular haemodynamics and hormonal responses in male dogs. By comparing the outcomes of hCG treatment across different seasons, this research seeks to provide a more nuanced understanding of how environmental factors might influence the efficacy of reproductive interventions.

## 2 Materials and Methods

### 2.1 Study Design and Subjects

The study was conducted with 12 clinically healthy male crossbreed dogs, aged 2–5 years. To evaluate seasonal variations, the study was carried out in four distinct seasons: spring, summer, fall and winter, in **Karaj, Alborz, Iran**. The average temperature ranges in Karaj during the study period were approximately **10°C–20°C in spring**, **25°C–40°C in summer**, **10°C–20°C in fall** and **−2°C to 8°C in winter**. The dogs were kept under standard kennel conditions, provided with commercial food and had free access to water. Their fertility status was confirmed through sperm analysis and sonographic examination.

### 2.2 Experimental Procedure

At the beginning of each season, baseline measurements for all parameters (hormonal levels and Doppler indices) were taken **before administering the hCG treatment**. These baseline evaluations served as **control measurements**, allowing for within‐subject comparisons. **For the control group, each dog received an injection of distilled water as the solvent used for the hCG dose**. A single intramuscular injection of hCG at a dose of 100 IU/kg (**Folignan 500U; Darou Pakhsh Pharma Chem Co., Tehran**) was then given after the initial evaluation. Injections were administered between 8:00 and 9:00 AM to control for circadian effects on blood flow. Measurements were repeated at multiple time points post‐injection to assess the effects of hCG compared to baseline. **This approach allowed us to use the pre‐injection values as a control, reducing the need for a separate control group** (Kobayashi, Hori, and Kawakami 2014). The same dogs were used in each seasonal study, with a minimum washout period of 3 months between trials to avoid any carryover effects.

### 2.3 Sample Collection

Blood samples (5 mL) were collected into heparinised tubes before hCG injection (baseline) and at 1, 3, 6, 12, 24, 48, 72, 96, 144 and 168 h post‐injection. Plasma was separated by centrifugation at 1500 g for 15 min and stored at −20°C until hormonal assays were performed.

### 2.4 Hormonal Analysis

Testosterone and oestrogen levels were quantified using specific ELISA kits. For testosterone, we used the **canine testosterone (T) ELISA Kit** (Catalogue Number: **CSB‐E06893c**; **Cusabio Biotech Co., Wuhan, China**). This kit has a detection range of **0.1–20 ng/mL** and a sensitivity of **0.05 ng/mL**. The intra‐assay and inter‐assay coefficients of variation (CVs) were both less than **15%**.

For oestrogen, we utilised the **canine oestrogen (competitive EIA) ELISA Kit** (Catalogue Number: **LS‐F39302**; **LifeSpan BioSciences, Seattle, WA, USA**). The detection range for this kit is **15.6–1000 pg/mL**, with a sensitivity of **15.6 pg/mL**. The intra‐assay CV was less than **8%**, and the inter‐assay CV was less than **10%**.

**Each determination was performed in triplicate** to ensure the accuracy and reliability of the results. The values from these replicates were averaged for final data analysis, minimising the potential for outliers or variability. In cases where significant variation between replicates was observed, the samples were re‐analysed to ensure accuracy.

### 2.5 Ultrasonography Protocol

**FIGURE 1:**
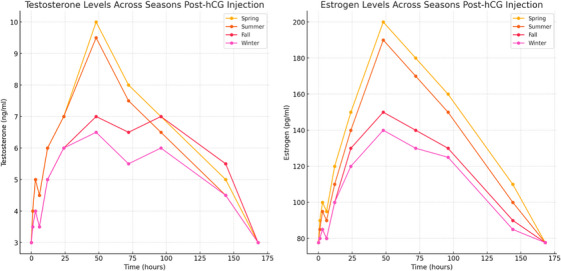
Colour Doppler ultrasonography shows blood flow within the convoluted part of the supra‐testicular artery (left side). Pulsed‐wave Doppler ultrasonography reveals a wave‐like pattern as monophasic non‐resistive waveforms of blood flow within the supra‐testicular artery (right side).

Testicular blood flow was assessed using a colour Doppler ultrasound system (Exago, France) with a 5–10 MHz linear probe. Measurements focused on the supra‐testicular artery (STA), with Doppler analysis performed to record resistive index (RI) and pulsatility index (PI). The procedure was consistent across all seasons, with evaluations conducted immediately after blood sample collection (Figure 1) (Hedia et al. 2020).

### 2.6 Statistical Analysis 

Data were analysed using repeated‐measures ANOVA to assess the effects of seasonality on hCG responses, followed by Student's *t*‐tests for post hoc comparisons. Pearson correlation analysis was conducted to evaluate the relationships between testicular Doppler parameters (RI and PI) and hormone levels (testosterone and oestrogen). Statistical significance was defined as *p* < 0.05.

## 3 Results

### 3.1 Seasonal Variations in Hormonal Response

**FIGURE 2:**
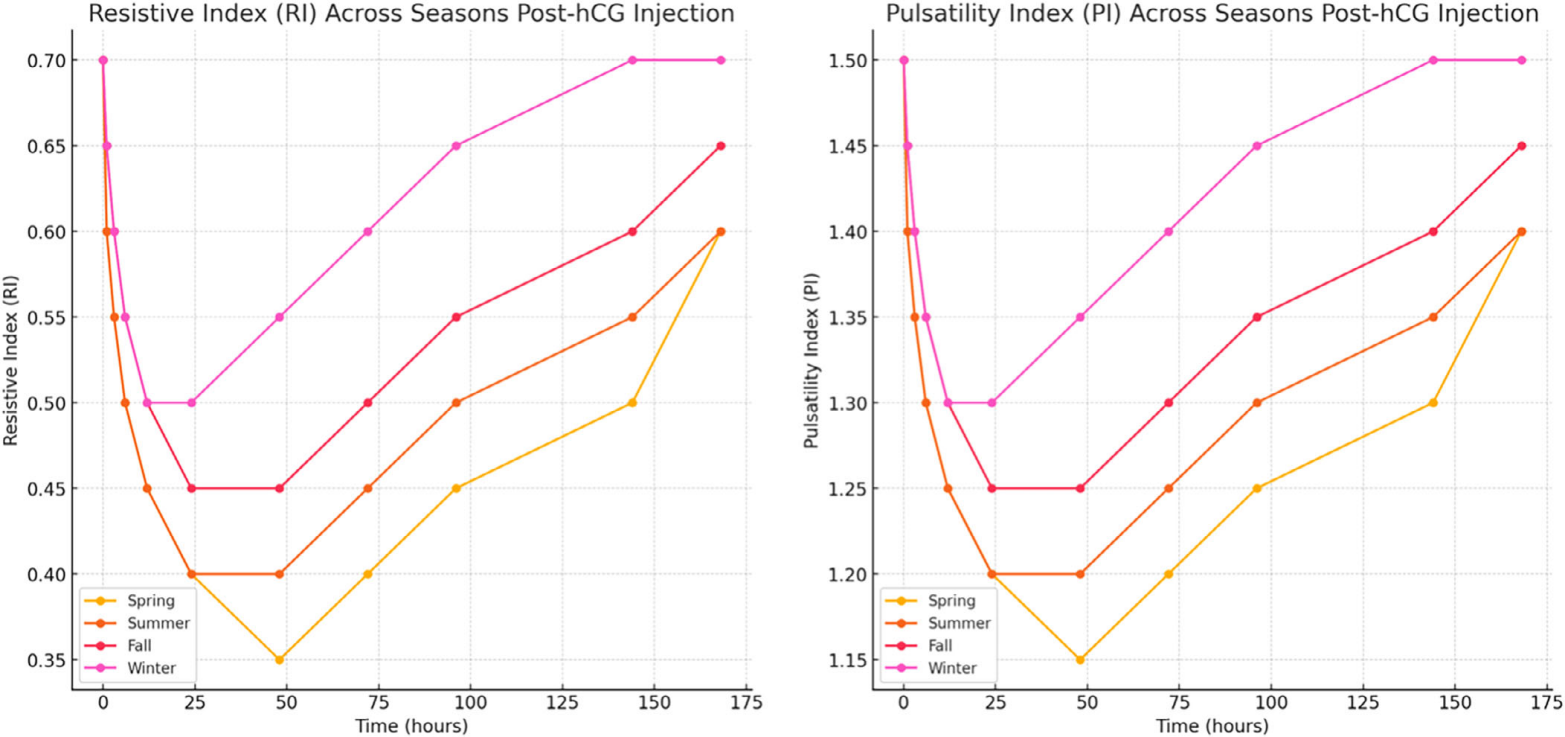
Seasonal variation in hormonal response to hCG injection. This figure shows testosterone (left) and oestrogen (right) levels over time after hCG injection in male dogs, comparing responses across spring, summer, fall and winter. The points represent the mean of three replicate measurements taken at each time point for each season. Higher hormone levels are seen in spring and summer, indicating a seasonal influence on endocrine response.

Testosterone levels exhibited significant seasonal variation following hCG injection. In spring and summer, testosterone levels peaked earlier (at 48 h) compared to fall and winter, where the peak was observed at 96 h. The magnitude of the increase was also greater in spring and summer, suggesting heightened responsiveness during these seasons. Oestrogen levels followed a similar seasonal trend, with a more pronounced increase in spring and summer compared to the cooler seasons (Figure 2).

### 3.2 Seasonal Impact on Testicular Blood Flow

**FIGURE 3:**
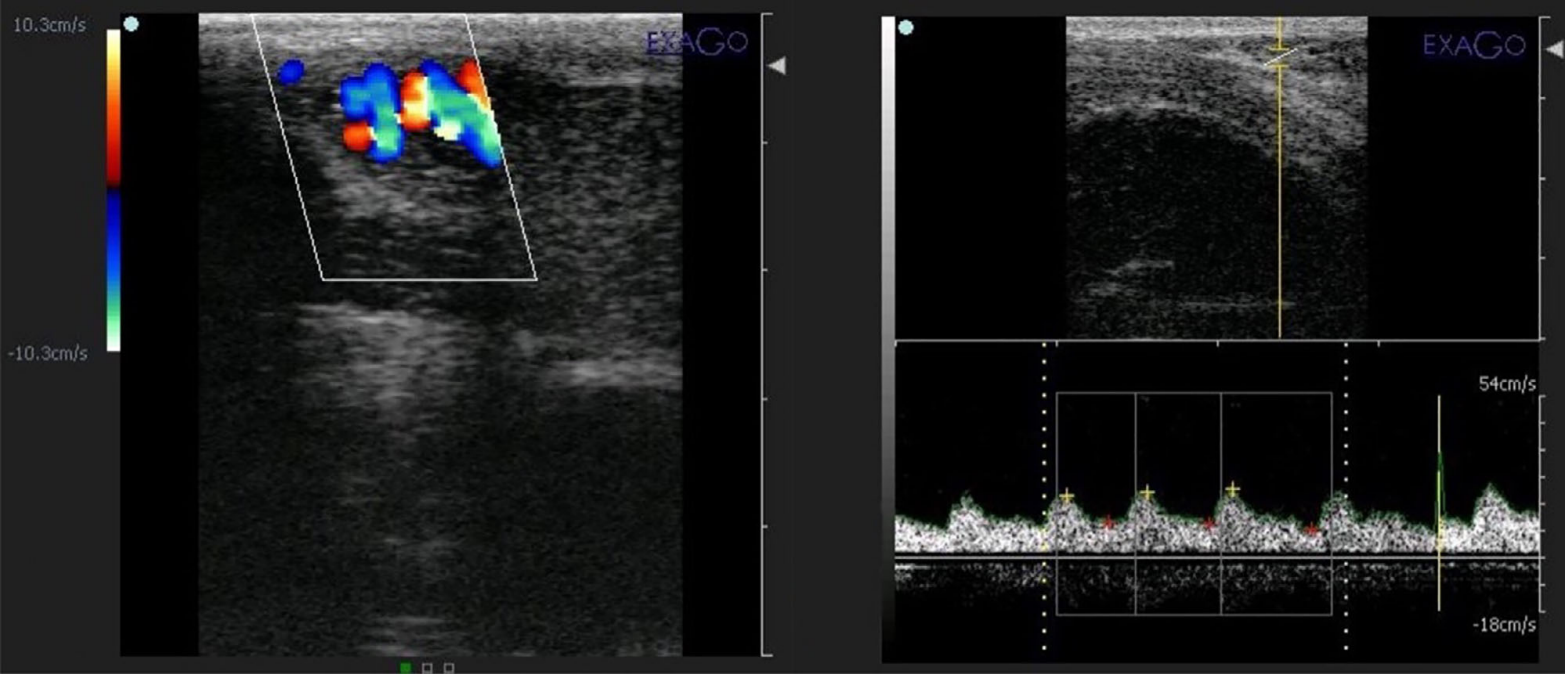
Seasonal impact on testicular blood flow indices post‐hCG injection. This figure displays resistive index (RI (left) and pulsatility index (PI) (right) across different seasons, measured after hCG injection. The data reveal improved testicular blood flow in spring and summer, highlighting seasonal effects on vascular response to treatment.

Doppler ultrasonography revealed that the RI decreased significantly in response to hCG across all seasons, indicating increased testicular blood flow. However, the extent of this decrease was most substantial in spring, followed by summer, with fall and winter showing less marked changes. PI also showed seasonal differences, with the most significant reductions occurring in spring and summer, suggesting improved vascular dynamics during these periods (Figure 3).

### 3.3 Seasonal Correlation Analysis

Pearson correlation analysis showed a moderate negative correlation between RI and testosterone levels (*r* ≈ −0.85) and between PI and testosterone (*r* ≈ −0.80) during spring and summer, indicating improved testicular blood flow was associated with higher testosterone. Correlations with oestrogen were weaker, with RI showing a weak negative correlation (*r* ≈ −0.60) and PI a moderate negative correlation (*r* ≈ −0.70). These results suggest a significant seasonal influence on the relationship between testicular haemodynamics and hormone levels, particularly testosterone, after hCG administration (Table 1).

**TABLE 1:** Seasonal correlation of Doppler indices and hormonal responses post‐hCG administration in canines.

Correlation	Correlation coefficient (*r*)	Seasonal influence
Testosterone and RI	−0.85	Spring/Summer
Testosterone and PI	−0.8	Spring/Summer
Oestrogen and RI	−0.6	Spring/Summer
Oestrogen and PI	−0.7	Spring/Summer

### 3.4 Conclusion of Results

The data clearly indicate that the physiological response to hCG is modulated by seasonal factors, with spring and summer providing a more favourable environment for hCG‐induced enhancements in testicular function. The differences in hormonal levels and blood flow parameters across seasons underscore the importance of considering environmental factors when planning reproductive treatments in canines.

## 4 Discussion

The results of this study highlight the significant impact of seasonality on the efficacy of hCG treatment in male dogs. The enhanced response observed in spring and summer, characterised by greater increases in testosterone and oestrogen levels and more pronounced improvements in testicular blood flow, suggests that environmental factors such as temperature, daylight length and perhaps even seasonal variations in metabolic activity play a crucial role in modulating reproductive physiology (Ortega‐Pacheco et al. 2006).

Previous studies in other species have shown that reproductive parameters such as sperm quality and testicular size vary with the seasons, often peaking during periods of optimal environmental conditions (Prochowska, Partyka, and Niżański 2021; Perumal et al. 2022; Sampaio et al. 2023; Suliman et al. 2020; Mandal, Kumar, and Tyagi 2022). Our findings align with these observations, indicating that hCG's effectiveness in enhancing reproductive function is also subject to seasonal modulation. However, the specific seasonal patterns observed in dogs suggest that canines may have unique responses to environmental changes, possibly due to their domestication and adaptation to a wide range of climates.

The use of colour Doppler ultrasonography provided valuable insights into the haemodynamic changes induced by hCG, revealing how these changes vary with season (Velasco and Ruiz 2020; Hedia et al. 2020; Samir et al. 2015). The significant reductions in RI and PI during spring and summer suggest that these seasons provide a more conducive environment for enhancing testicular perfusion, potentially leading to improved spermatogenesis and fertility outcomes (Samir, Radwan, and Watanabe 2021; Ribeiro et al. 2020; Abdelnaby 2022; Rawy et al. 2021).

The correlation analysis revealed a strong negative relationship between testicular blood flow (RI and PI) and testosterone levels, particularly in spring and summer, indicating that increased blood flow is linked to higher testosterone production. Weaker correlations were observed with oestrogen, suggesting that testosterone plays a more dominant role in the seasonal modulation of reproductive function. These findings highlight the importance of testicular perfusion in regulating hormonal responses during periods of increased reproductive activity.

The seasonal variations in response to hCG observed in this study have important implications for veterinary practice, particularly in the management of canine infertility (Hien 2020). Understanding that the efficacy of hCG treatment may be enhanced during certain seasons can inform the timing of therapeutic interventions, potentially improving outcomes for dogs with reproductive issues. The **spring and summer** seasons appear to offer optimal conditions for hCG‐induced improvements in **testicular function**, emphasising the need for seasonally timed treatment strategies.

## 5 Conclusion

This study provides compelling evidence that seasonality plays a significant role in modulating the physiological effects of hCG on testicular function in male dogs. The enhanced hormonal response and improved blood flow during spring and summer highlight the importance of considering environmental factors when administering reproductive treatments. Future research should explore the underlying mechanisms driving these seasonal variations and assess the long‐term effects of seasonally timed hCG treatment on fertility inx canines.

## Author Contributions

**Behnam Nadali**: conceptualisation (lead), investigation (lead), methodology (equal), writing–original draft (equal). **Arman Abdous**: conceptualisation (equal), formal analysis (lead), investigation (supporting), project administration (lead), writing–review and editing (equal). **Nima Karami**: data curation (lead), resources (equal), validation (equal), writing–review and editing (supporting). **Mohammad Jokar**: investigation (equal), resources (supporting), writing–review and editing (supporting). **Ourang Ataie Amarloie**: supervision (lead), validation (supporting), writing–review and editing (equal). **Mehran Farhoodi**: data curation (supporting), methodology (equal), writing–review and editing (equal).

## Ethics Statement

All experimental procedures were approved by the Ethical Review Committee of the Karaj Azad University Department of Clinical Sciences and adhered to the guidelines of the Iranian Society for the Prevention of Cruelty to Animals.

## Conflicts of Interest

The authors declare no conflicts of interest.

### Peer Review

The peer review history for this article is available at https://publons.com/publon/10.1002/vms3.70117.

## Data Availability

The data supporting the findings of this study are available from the corresponding author upon reasonable request.
